# Monosodium Glutamate (MSG) Consumption Is Associated with Urolithiasis and Urinary Tract Obstruction in Rats

**DOI:** 10.1371/journal.pone.0075546

**Published:** 2013-09-26

**Authors:** Amod Sharma, Vitoon Prasongwattana, Ubon Cha’on, Carlo Selmi, Wiphawi Hipkaeo, Piyanard Boonnate, Supattra Pethlert, Tanin Titipungul, Piyapharom Intarawichian, Sakda Waraasawapati, Anucha Puapiroj, Visith Sitprija, Sirirat Reungjui

**Affiliations:** 1 Department of Biochemistry, Faculty of Medicine, Khon Kaen University, Khon Kaen, Thailand; 2 Rheumatology and Clinical Immunology, Humanitas Clinical and Research Center, Milan, Italy; 3 BIOMETRA Department, University of Milan, Milan, Italy; 4 Department of Anatomy, Faculty of Medicine, Khon Kaen University, Khon Kaen, Thailand; 5 Department of Pathology, Mahasarakham Hospital, Mahasarakham, Thailand; 6 Department of Pathology, Faculty of Medicine, Khon Kaen University, Khon Kaen, Thailand; 7 Queen Saovabha Memorial Institute, Bangkok, Thailand; 8 Department of Internal Medicine, Faculty of Medicine, Khon Kaen University, Khon Kaen, Thailand; University of Sao Paulo Medical School, Brazil

## Abstract

**Background:**

The peritoneal injection of monosodium glutamate (MSG) can induce kidney injury in adult rats but the effects of long-term oral intake have not been determined.

**Methods:**

We investigated the kidney histology and function in adult male Wistar rats that were fed *ad* *libitum* with a standard rat chow pellet and water with or without the addition of 2 mg/g body weight MSG/day in drinking water (n=10 per group). Both MSG-treated and control animals were sacrificed after 9 months when renal function parameters, blood and urine electrolytes, and tissue histopathology were determined.

**Results:**

MSG-treated rats were more prone to kidney stone formation, as represented by the alkaline urine and significantly higher activity product of calcium phosphate. Accordingly, 3/10 MSG-treated rats developed kidney stones over 9 months versus none of the control animals. Further, 2/10 MSG-treated rats but none (0/10) of the controls manifested hydronephrosis. MSG-treated rats had significantly higher levels of serum creatinine and potassium including urine output volume, urinary excretion sodium and citrate compared to controls. In contrast, MSG-treated rats had significantly lower ammonium and magnesium urinary excretion.

**Conclusion:**

Oral MSG consumption appears to cause alkaline urine and may increase the risks of kidney stones with hydronephrosis in rats. Similar effects in humans must be verified by dedicated studies.

## Introduction

Monosodium glutamate (MSG) is used as a flavor enhancer in food preparation to increase its palatability [1,2] and the MSG consumption is growing worldwide with average daily intake estimated as 3-4 g/day [3-5]. The Food and Drug Administration (FDA) has determined MSG as safe for the general population and accordingly stated that an Acceptable Daily Intake (ADI) is not specified [1,6]. However, recent studies support the hypothesis that MSG consumption may be associated with overweight, the metabolic syndrome, or arterial hypertension [3-5,7] albeit these conclusions remain debated [8,9]. No data are available on the effects of MSG on the urinary apparatus and kidney function in humans. Of note, chronic oral MSG intake in rats leads to changes in antioxidant systems and renal markers including lipid peroxidation byproducts [10], in agreement with what observed in rats injected with MSG [11]. Moreover, dietary MSG increases the urinary pH in rats [12] and alkaline urine may influence the kidney capacity to secrete or reabsorb metabolites that contribute to stone formation, as in the case of calcium phosphate products [13]. Based on these observations we hypothesized that chronic oral MSG consumption may increase the risk of renal stone formation and renal function impairment and the aim of the present study is to investigate the nephrological effects of long-term oral MSG consumption in rats. 

## Materials and Methods

### Chemicals and animals

MSG used in this study was a commercially available 99%-pure food-grade package, while all other chemicals and solvents were of analytical grade. Adult male Wistar rats (150-200g) were obtained from the National Laboratory Animal Center (Salaya, Mahidol University, Bangkok, Thailand) and housed under controlled laboratory conditions of temperature (25 ± 2 °C), humidity (60%) and 12 h light/12 h dark cycle. All experiments were done under the guideline of the Northeast Laboratory Animal Center (NELAC), Khon Kaen University, Thailand, and approved by the Animal Ethics Committee of Khon Kaen University, Thailand.

### Experimental design

A group of 6-week old rats (n=20) received a standard rat chow pellet (Perfect Companion Group, Bangkok, Thailand) and water *ad libitum*. In the treated group (n=10), MSG was added to drinking water to achieve a daily dose of 2 mg/g body weight as estimated by daily water intake measurements. Mean MSG consumption was calculated from the amount of MSG water consumed daily, and expressed in mg per gram of body weight. The MSG concentration in drinking water based on the weekly water intake and previous body weight ranged between 0.6%-2.0% during the 9 months of the study. Food intake and body weight were recorded every one and two weeks, respectively. Animals of both groups were sacrificed 9 months later by intraperitoneal Nembutal injection (euthanizing dose) after 12-hour fasting. Urine samples were collected one week before sacrifice. The animals were housed in individual stainless steel metabolic cages designed for the separate collection of urine and a 24-hour urine sample was collected from each animal. Urine volumes were recorded and samples were kept at −70°C until analyzed. Blood samples were collected on the day of sacrifice by abdominal venous puncture and serum and plasma were separated by centrifugation at 1500xg, 4°C for 10 minutes and stored at −70°C until analyzed. Kidneys were washed with normal saline, dissected, and fixed in 4% paraformaldehyde solution for histopathological analysis.

### Histology

Routinely processed paraffin-embedded tissue blocks were sliced at 4 µm thickness. Sections were stained with Haematoxylin & Eosin (H&E) and observed under a light microscope (Primo Star, Zeiss). To determine the percentage of fibrosis, kidney sections were stained with Masson’s Trichrome, scanned by ScanScope slide scanner (Aperio) and interpreted using Aperio ImageScope Version 11.1.2.752. Glomeruli were excluded and the remaining areas were analyzed by positive pixel count protocol; blue areas of Masson staining were counted as negative pixel. The fibrotic results were calculated by negative pixel x100 divided by total pixels. Analysis of renal sections for stone crystals was done under a polarized light microscope (Olympus BX 51).

### Serum and urine biochemistries

Serum creatinine and blood urea nitrogen (BUN) concentrations were determined by Jaffe [14] and diacetyl monoxime [15] methods, respectively. Inorganic phosphate concentration was determined as described elsewhere [16]. Urinary analyses included the measurements of ammonium by Chaney et al. 1962 [17], calcium and magnesium by atomic absorption [18], and protein by Lowry’s method [19]. Serum and urine electrolytes were analyzed by auto analyzer (Automate Cobas C6000). Citrate in urine was estimated by a new citrate lyase method [20]. Based on the urinary excretion of calcium, citrate, phosphate, urine pH and the urine volume, we then calculated the ion-activity products of calcium phosphate [AP(CaP)] [21]. The index estimates the risk of *in vivo* crystalization of CaP in urine using the following formula:

$$\text{AP(CaP)-index}=\frac{2.7\times 10^{-3}\times {\text{Ca}}^{1.07}\times {\text{P}}^{0.7}\times {\left(\text{pH}-4.5\right)}^{6.8}}{{\text{Citrate}}^{0.20}\times {\text{V}}^{1.31}}$$

### Statistical Analysis

Continuous variables are expressed as mean ± standard deviation and differences between groups were compared for statistical significance by Student *t*-test. All comparisons were two-tailed and *P* values < 0.05 were considered statistically significant. 

## Results

### Growth, food, water, MSG, and Na intake

**Figure 1:**
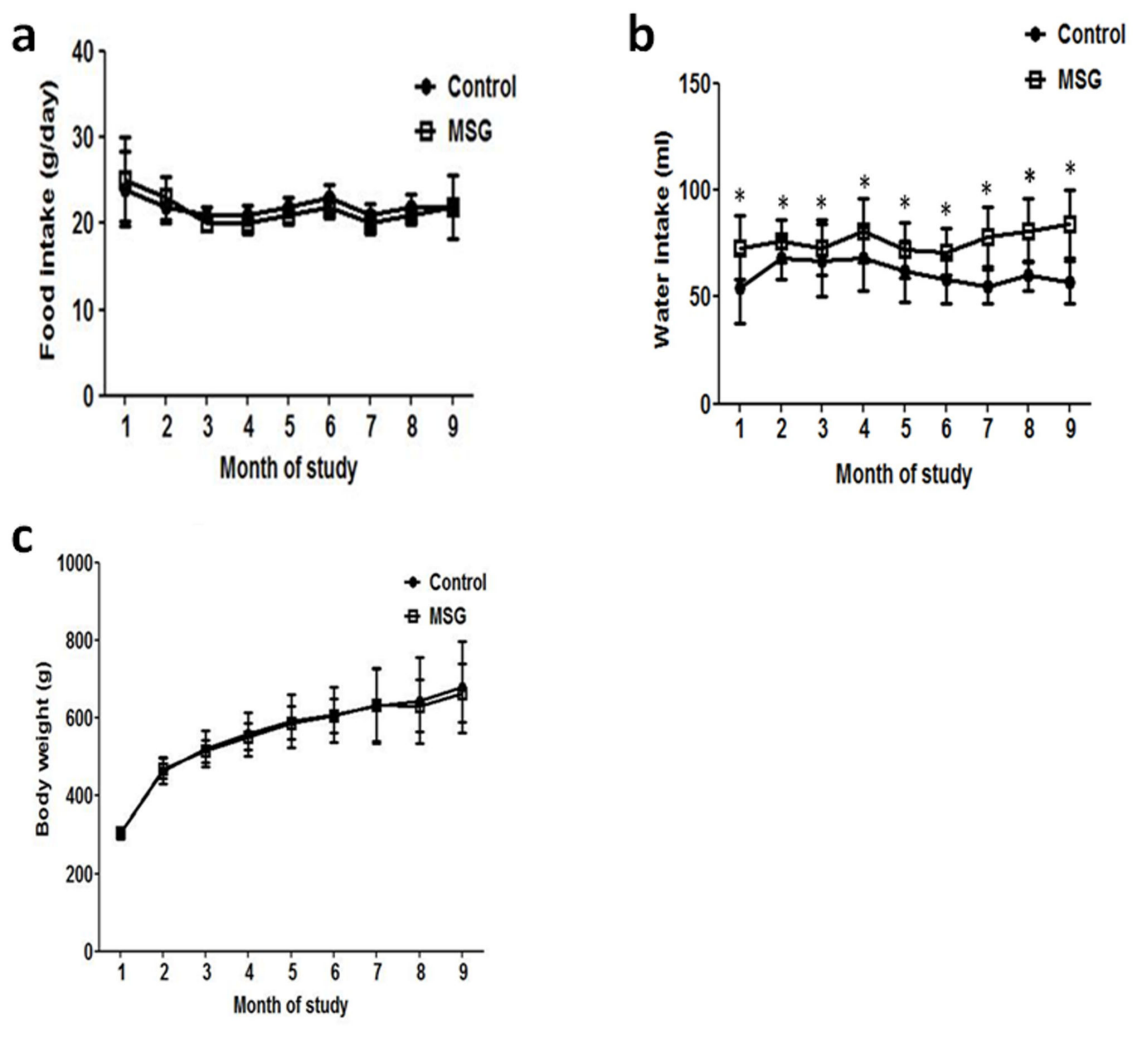
Mean food intake (a), water intake (b), and growth curve (c) in 10 rats from the MSG-treated and control groups at different time-points over 9 months.

Animals allocated to the MSG or control groups had similar daily food intake, body weight and kidney weight at the end of the study (Figure **1a, 1c** and Table **1**). On the other hand, MSG-treated rats had a significantly higher daily water intake compared to controls throughout the study (Figure **1b**). Based on the similar daily food intake (22g/day) and the provided food containing 0.2 g% Na, all rats received 0.044 g/day Na from regular chow. Further, in MSG-treated rats the mean ± SD daily MSG intake was 2.10 ± 0.32 mg/g body weight throughout the study corresponding to 0.29 ± 0.04 mg/g bw/day (0.19 g/day) Na from MSG supplementation.

**Table 1:** Body weight, water, food intake, and kidney weight in control and MSG-treated rats at 9 months.

	**Control (n=10)**	**MSG (n=10)**
Baseline (6 weeks) body weight (g)	204.3 ± 12.1	204.7 ± 12.5
Final body weight (g)	680 ± 118	665 ± 75
Daily water intake (ml)	57 ± 10	84 ± 16^*^
Daily food intake (g)	22 ± 3.7	22 ± 0.5
Kidney weight (g)	1.42 ± 0.17	1.50 ± 0.24

Variables are expressed as mean ± standard deviation. *p < 0.05

### Kidney pathology

**Figure 2:**
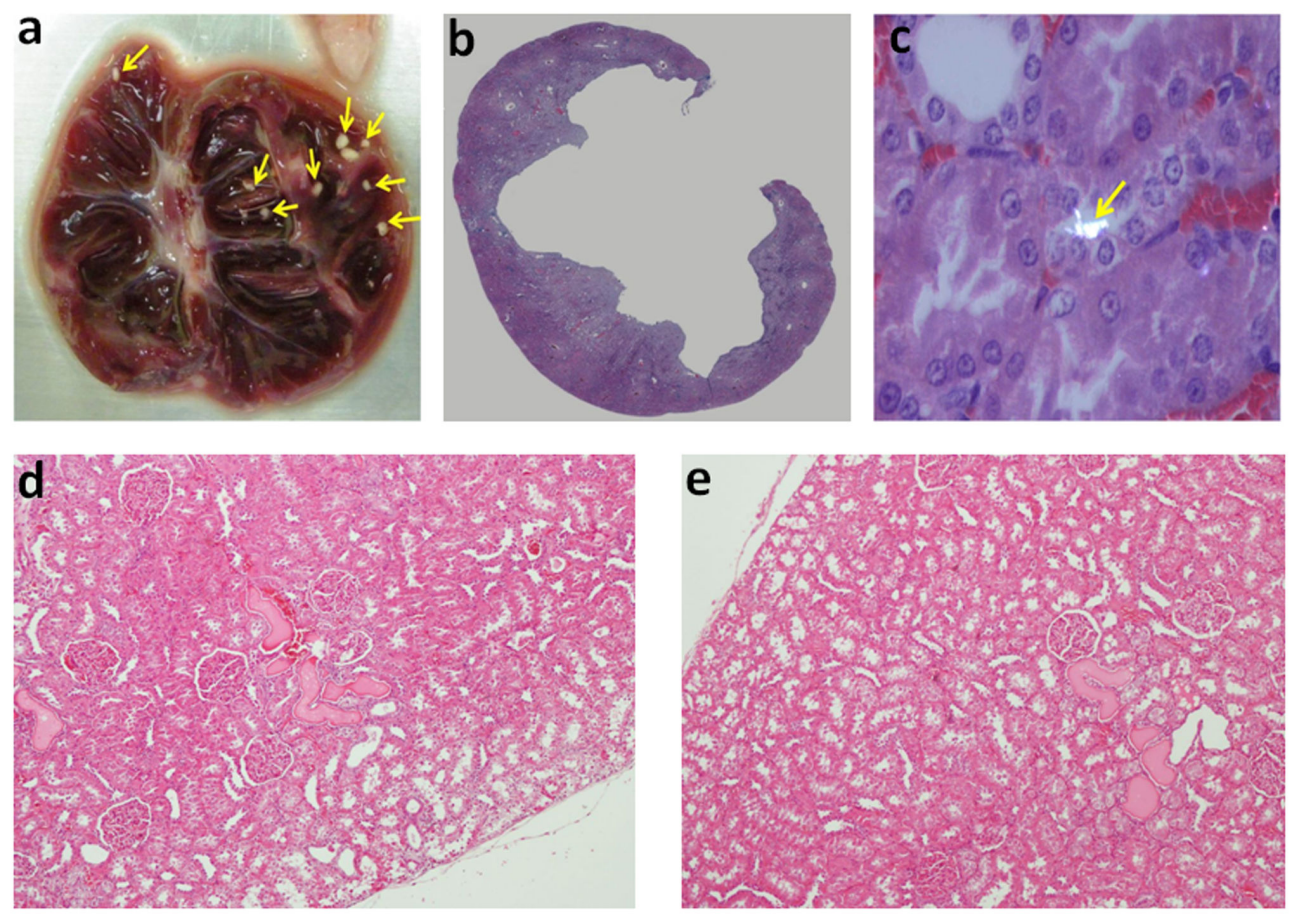
Macroscopic appearance of one representative case of urolithiasis in the MSG group (a). Virtual scanning of H&E kidney sections of MSG treated animal with moderate hydronephrosis (5x) in 9 months (b). Crystal in the kidney tissue under dark field illumination with polarized microscope (Olympus BX51) in 9 months MSG-treated animal (200X) (c). H&E staining revealed the hyaline casts including the flattening of the tubular epithelium in both control (d) and MSG-treated tissues (e) (100x).

**Figure 3:**
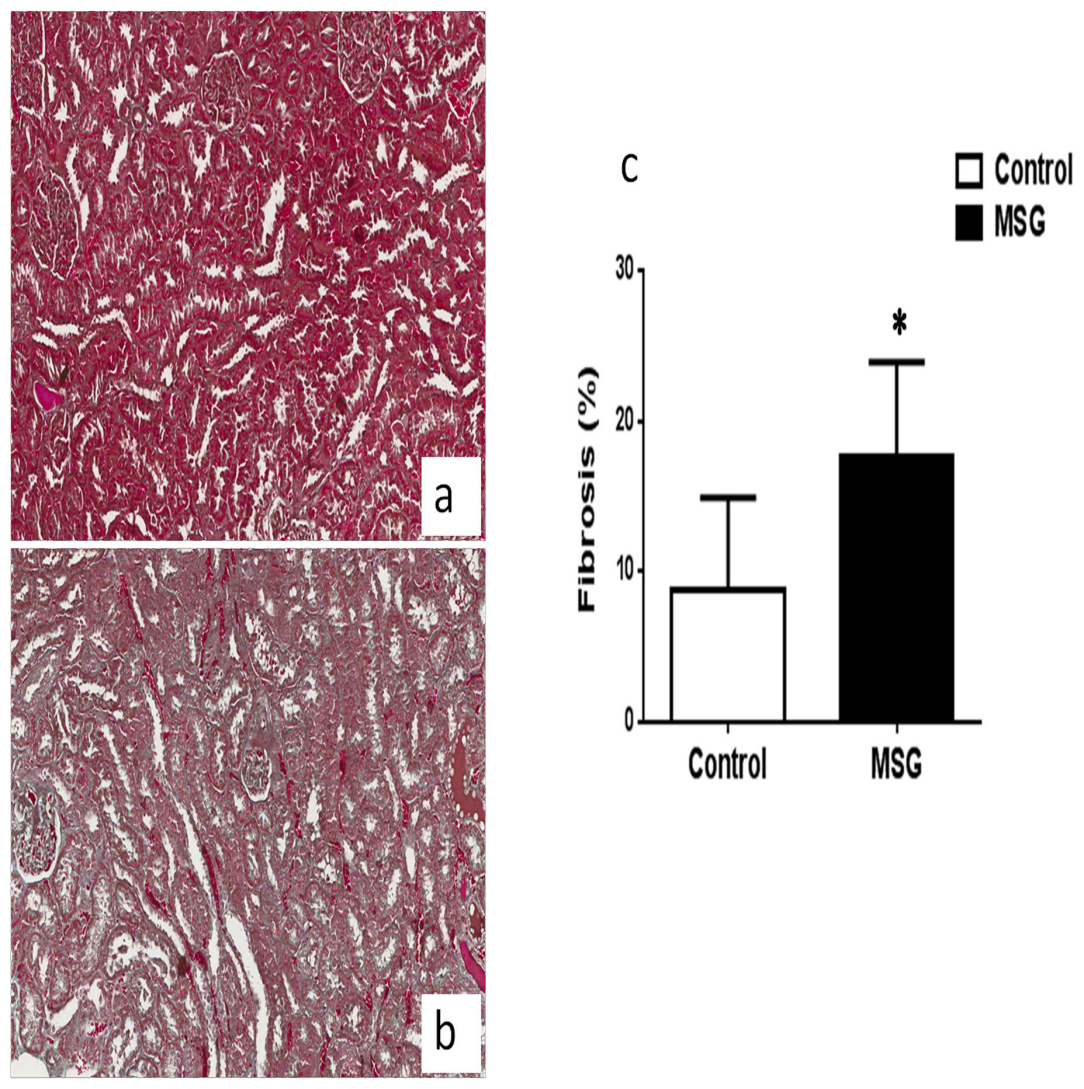
Renal interstitial fibrosis in 9 months MSG-treated animals (100x).

The kidney gross anatomical examination revealed the presence of lithiasis in 3/10 MSG-treated rats and 0/10 controls. Kidney stones were minute, white in color with smooth surface, numerous in numbers and localized in the calyx and pelvis area (Figure **2a**). In 2 of the 3 cases the lithiasic kidneys manifested flattened and atrophic renal papillae out of proportion to the cortex, which is the characteristic feature of hydronephrosis (Figure **2b**). Intra-luminal white crystals were also observed in the kidney tissue of 2/10 treatment animals but 0/10 controls under polarized microscope supporting pre-lithiasis (Figure **2c**). It should be noted that one of the animals with intra-luminal crystal is not the same animal with renal stone. Further histological examination revealed the hyaline casts including the flattening of the tubular epithelium in both groups at similar levels (Figure 2d, 2e). By histological quantification, the kidneys of MSG-fed rats had significantly increased interstitial fibrosis (17.6 ± 6.3%) compared to those of controls (8.7 ± 6.2%) (Figure **3**).

### Blood and urine biochemistries

**Figure 4:**
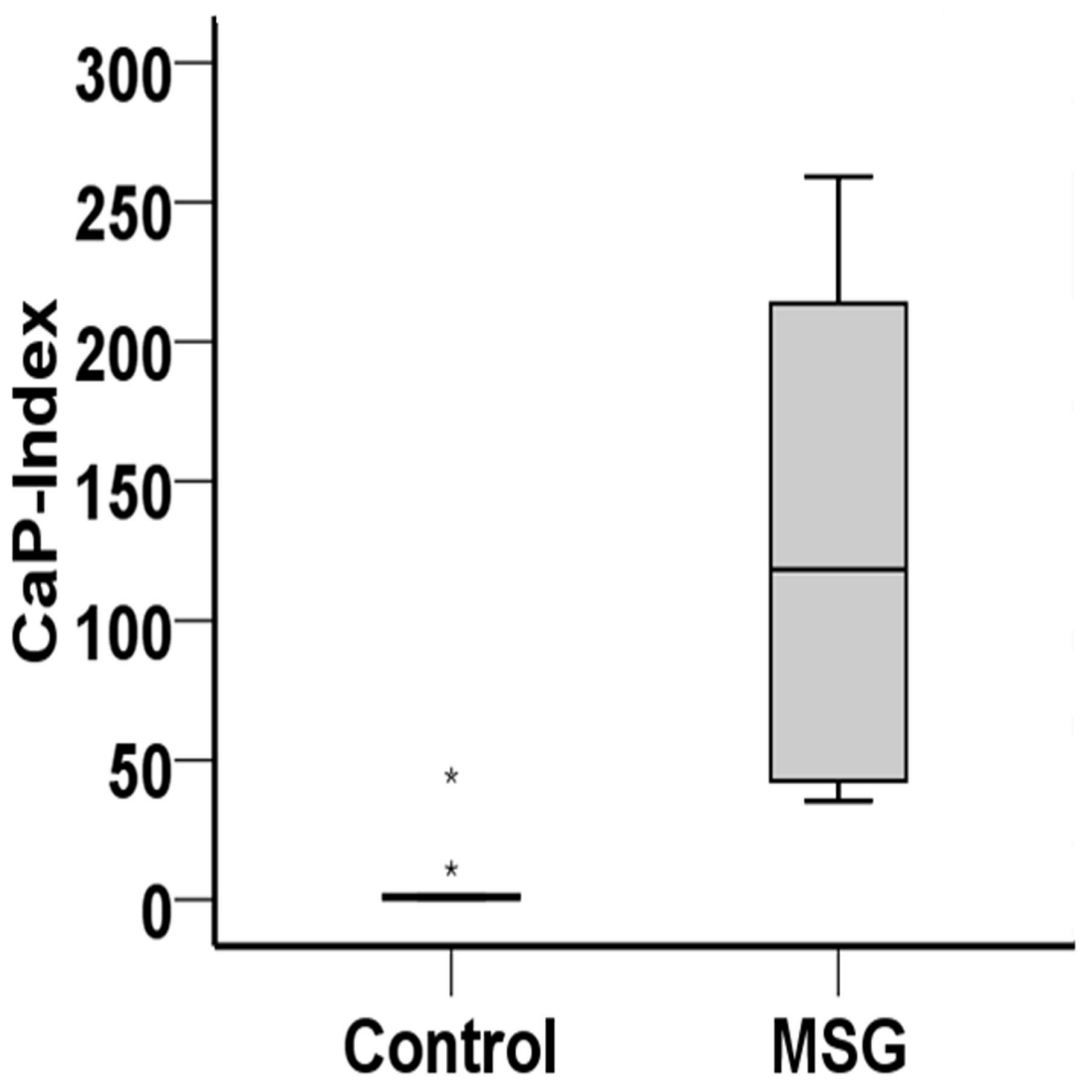
CaP-index in 9 months control and MSG-treated rats.

Urine analyses demonstrated that MSG-treated rats had significantly higher urine pH and urine output compared to controls (Table **2**). The daily urinary excretion of sodium and citrate were also significantly higher in the MSG group while the amount of urinary ammonium and magnesium were significantly lower compared to the control group. Most notably, MSG treated-group had significantly higher AP(CaP) value (135.77 ± 124.46) compared to controls (6.76 ± 6.01) (Figure **4**). There were no significant differences in the urine excretion of potassium, chloride, calcium and phosphate between groups. At the end of the study period, serum creatinine was significantly higher in the MSG group compared to controls (Table **3**). Among blood electrolytes, serum potassium levels were significantly higher in MSG-treated rats than in controls, but all other electrolytes (Na^+^, Cl^-^, HCO_3_^-^, Ca^2+^, PO_4_^2-^, Mg^2+^) were comparable between both groups.

**Table 2:** Urine biochemistry in control and MSG-treated rats at 9 months of study.

	**Control (n=10)**	**MSG (n=10)**	**P value**	
Urine Volume (ml/day)	26.9 ± 7.2	51.5 ± 23.6	< 0.01	
Urine pH	6.6 ± 0.6	8.7 ± 0.4	< 0.001	
Sodium (mEq/day)	1.0 ± 0.5	6.1 ± 2.9	< 0.001	
Potassium (mEq/day)	4.1 ± 1.2	4.8 ± 1.5	NS	
Chloride (mEq/day)	1.3 ± 0.6	1.8 ± 0.7	NS	
Calcium (mg/day)	3.9 ± 1.2	4.1 ± 1.2	NS	
Phosphate (mg/day)	36 ± 15	39 ± 5	NS	
Citrate (mmol/day)	0.43 ± 13	0.77 ± 0.16	< 0.001	
Ammonium (mmol/day)	1.41 ± 0.39	0.38 ± 0.19	< 0.001	
Magnesium (mg/day)	4.1 ± 0.9	2.0 ± 1.1	< 0.001	

Variables are expressed as mean ± standard deviation. NS, not significant.

**Table 3:** Serum biochemistry in control and MSG-treated rats at 9 months of study.

	**Control (n=10)**	**MSG (n=10)**	**P value**
BUN (mg/dL)	23.3 ± 3.1	22.4 ± 3.2	NS
Creatinine (mg/dL)	1.42 ± 0.11	1.60 ± 0.07	< 0.001
Sodium (mEq/L)	144.9 ± 1.6	145 ± 1.5	NS
Potassium (mEq/L)	4.4 ± 0.6	5.3 ± 1.1	< 0.05
Bicarbonate (mEq/L)	21.1 ± 3.2	21.8 ± 2.9	NS
Chloride (mEq/L)	102.9 ± 1.5	103.7 ± 1.8	NS
Calcium (mEq/L)	10.3 ± 0.4	10.5 ± 0.3	NS
Magnesium (mEq/L)	2.3 ± 0.2	2.5 ± 0.4	NS
Phosphate (mEq/L)	4.9 ± 1.3	5.2 ± 0.9	NS

Variables are expressed as mean ± standard deviation. NS, not significant.

### Stone composition

Regarding a small quantity of collected stones, only the calcium level can be determined. Using the atomic absorption method, the level of calcium was approximately 1 ppm.

## Discussion

MSG is gaining a central role as a flavor enhancer in modern-day cuisine, particularly of Asian Pacific countries. At present, an acceptable daily intake for MSG in humans remains undetermined and intakes of 0-120 mg/kg body weight were suggested in 1974 by the WHO. Accordingly, the Scientific Committee for Food (SCF) at the European Commission mentioned in 1991 that MSG is associated with an “ADI not specified” and this remains the current indication in the European Union. However, a dose of 150 mg/kg body weight has been mentioned as safe [6] and has been tested in healthy volunteers [22] while in our previous study some of the subjects also consumed MSG in the same range (0.4-14 g/day) [5]. Based on dose translation between rodents and humans [23], the MSG dose used in the present study is 22.7 g/day for a 70 kg man which is approximately 2-3 times higher than the safe dose that has been used in healthy volunteers [22] and 5-6 times higher than the current reported consumption in Asian Countries [3-5].

The association between dietary factors, including MSG, and the risk of kidney disease has been proposed in numerous studies without reaching solid conclusions. In previous experiments, MSG supplementation either by injection [11] or oral intake [10,24] induced kidney damage but we report herein for the first time that the chronic consumption of dietary MSG causes obstructive nephropathy from urolithiasis in adult rats possibly via urine alkalinization which predispose to CaP precipitation.

It should be noted that all MSG-treated rats had alkaline urine. We suspected that MSG-treated animals may generate higher catabolic products of glutamate in kidney cells and its carbon skeleton is converted into carbon dioxide (CO_2_) and then to bicarbonate anion [25-27]. The generated bicarbonate is then absorbed back to blood circulation and ultimately to kidney for excretion of the extra-alkali, exhibiting alkaline urine [28,29]. Despite the unknown mechanism by which oral MSG increases urinary pH, urine analysis of MSG-treated rats such as significantly higher excretion of citrate but lower excretion of ammonium resemble what is observed in alkali-loaded animals [30]. This should be a renal compensation mechanism to neutralize alkali by lowering the excretion of ammonium ion (NH_4_^+^), while elevating the excretion of organic anion such as citrate during the alkali load [30-32]. The significant decrease in magnesium excretion found in MSG-treated rats may also be secondary to alkaline urine or alkali loading, consistent with a previous study that a decrease of Mg excretion was found in bicarbonate treated animals [33].

Alkaline urine has also been reported in rats receiving 6% dietary MSG for 3 months [12] and a similar observation was found in the present study with a 3-fold lower dose. It has been noted that urine pH exceeding 6.8 enhances the precipitation of urine constituents like CaP by 3-fold [34]. In agreement with our finding that MSG-treated rats had significantly higher value of AP(CaP) compared to controls. An increase of AP value indicated the risk of calcium-phosphate stone formation [21]. Based on the microscopic examination revealing intra-tubular crystals and the detectable calcium level in stone samples including alkaline urine, we are convinced that the stone found in MGS-treated rats is composed of calcium phosphate.

Urinary tract obstruction secondary to stones caused hydronephrosis in a subgroup of MSG-treated rats and the impaired renal function may result from the tubular damage and obstruction, thus causing renal dysfunction represented by the increase in serum creatinine and possibly potassium in MSG-treated rats. A similar degree of serum creatinine level increase had been previously reported following the oral exposure to a significantly higher amount of MSG [10].

Hydronephrosis was observed only in MSG-treated rats but not in control animals. Such moderate hydronephrosis resembles the effects of partial ureteral obstruction in rats [35] and obstructive nephropathy causes major changes in the tubulo-interstitial compartment of the kidney and renal interstitial fibrosis is a common long-term consequence [36]. We are convinced that this phenomenon may cause the kidney interstitial fibrosis observed in MSG-treated rats while oxidative stress secondary to MSG may also contribute [10] as reactive oxygen species induce the transmodulation of fibroblasts to myofibroblasts [37]. Tubular interstitial fibrosis is the strongest morphological predictor of clinical outcome in kidney disease, and is tightly associated to the progression of renal diseases [38].

We are aware of specific issues that may strengthen our findings or limit the impact of the data. First and foremost, both groups were given standard rodent diet and water *ad libitum* throughout the 9-months of observation, with the addition of MSG in drinking water as the sole difference. As expected, the MSG dose used in this study did not affect the growth and development of rats nor the daily food intake, as reported by Collison and Colleagues [39,40]. The total amount of MSG (0.6-2%) added in drinking water in this study is within the 0.2-8% dose range used in previous studies [10,41]. Second, the lithogenic effect of MSG may have been attenuated by the increased water consumption by MSG-treated rats, as well as the increased urinary citrate levels which counteract stone formation. Third, it should be noted that Wistar rats are susceptible to age-related nephropathy [42] and it has been reported that by 6 months of age, Wistar rats show minimal changes with scattered sclerotic glomeruli, a few foci of tubules dilated with eosinophilic (hyaline) casts and small mononuclear cell infiltrates in the interstitial tissue [43]. We also found features such as hyaline casts, and flattening of tubular epithelium in both control and MSG-treated groups. As a result, we cannot rule out the possibility that the strain susceptibility and aging may have concurred to the onset of kidney disease. Nevertheless, obstructive nephropathy had not been reported before in older rats.

Fourth, the standard rat chow used in this study had 24% protein content which may have some causal link with observed kidney pathology. It has been reported that protein-rich diets may cause a marked increase in water consumption, urine production and proteinuria [42]. We hypothesize that the increase in water consumption by MSG-treated rats may be secondary to MSG itself and not the high protein consumption since control and MSG-treated rats received the same amount of dietary protein. Further, the increase in water consumption of rats with a high protein diet was observed after 15 months [42] while the increased urine output found in both studies is likely proportional to water intake. As control rats had no obstructive nephropathy despite receiving similar amounts of protein, it is less likely that the obstructive nephropathy found in MSG-treated rats is related to the high protein diet.

Finally, features of MSG-treated rats resemble that of animals with a sodium-rich diet as increased water intake, high urinary sodium level, and fibrosis were reported also in Sprague Dawley rats receiving 8% NaCl diet for 3 weeks [44]. However, we are convinced that the changes observed in the present study do not recapitulate sodium enrichment. Indeed, sodium-loading animals manifested elevated calcium excretion that was not observed in MSG-treated rats and 8-week sodium intake was not associated with changes in serum creatinine and electrolyte (Na, K, Cl, Ca, Mg, P) levels [45], different from MSG-treated rats. Furthermore, the significant higher of urinary Ca, Cl, and Mg excretion found in sodium-loading rats is also opposite of what was observed in MSG-treated rats [45]. Most importantly, the total sodium intake in MSG-treated rats (0.32 g/kg) did not exceed the daily requirement (0.5 g/kg) [46] and the 24-hour sodium urinary excretion was within the normal range.

## Conclusions

Our data support the hypothesis that high doses of oral MSG cause urinary alkalinization and CaP saturation in rats thus possibly predisposing to kidney stone formation with subsequent hydronephrosis and interstitial fibrosis. While we are aware that the dosages employed in this study are in the higher range, albeit not infrequent in Asian real-life, we encourage human studies to observe whether this association is confirmed.
